# Supplementary material for: Rare genera differentiate urban green space soil bacterial communities in three cities across the world
Source: Access Microbiol. 2022 Jan 27;4(1):000320. doi: 10.1099/acmi.0.000320 (PMC8895604; doi:10.1099/acmi.0.000320)
Supplement: Supplementary material 1 [file acmi-4-0320-s001.pdf]

Rare genera differentiate urban green space soil bacterial communities in three cities across the world

Jacob G. Mills[1], Caitlin A. Selway[1], Laura S. Weyrich[1,2], Chris Skelly[3,4], Philip Weinstein[1,5,6], Torsten Thomas[7], Jennifer M. Young[1,8], Emma Marczylo[9], Sudesh Yadav[10], Vijay Yadav[10,D], Andrew J. Lowe[1,5], Martin F. Breed[1,5,8]

[1] School of Biological Sciences, The University of Adelaide, Adelaide, Australia

[2] Department of Anthropology and Huck Institutes of the Life Sciences, Pennsylvania State University, USA

[3] Healthy Urban Microbiome Initiative

[4] Research & Intelligence, Public Health Dorset, Dorset County Council, UK

[5] Environment Institute, The University of Adelaide, Adelaide, Australia

[6] School of Public Health, The University of Adelaide, Adelaide, Australia

[7] Centre for Marine Science and Innovation, School of Biological, Environmental and Earth Sciences, University of New South Wales, Sydney, Australia

[8] College of Science and Engineering, Flinders University, Bedford Park, South Australia

[9] Toxicology Department, Centre for Radiation, Chemical and Environmental Hazards, Public Health England, Chilton, Oxfordshire, UK.

[10] School of Environmental Sciences, Jawaharlal Nehru University, New Delhi, India

[D] Deceased

Corresponding author: Jacob G. Mills jacob.mills@adelaide.edu.au

Table S1 Bacterial 16S rRNA V4 region forward and reverse primers.

Primer	Primer sequence	Citations
515F	AATGATACGGCGACCACCGAGATCTACAC TATGGTAATT GT GTGCCAGCMGCCGCGGTAA	Caporaso et al. (2011); Caporaso et al. (2012)
806R X-barcode	CAAGCAGAAGACGGCATACGAGAT XXXXXXXXXXXX AGTCAGTCAG CC GGACTACHVGGGTWTCTAAT	

Table S2 Custom sequencing primers.

Read 1 sequencing primer	Primer sequence
Forward primer pad	TATGGTAATT
Forward primer linker	GT
Forward primer	GTGYCAGCMGCCGCGGTAA
Read 2 sequencing primer	Primer sequence
Reverse primer pad	AGTCAGCCAG
Reverse primer linker	CC
Reverse primer	GGACTACNVGGGTWTCTAAT
Index sequencing primer	AATGATACGGCGACCACCGAGATCTACACGCT

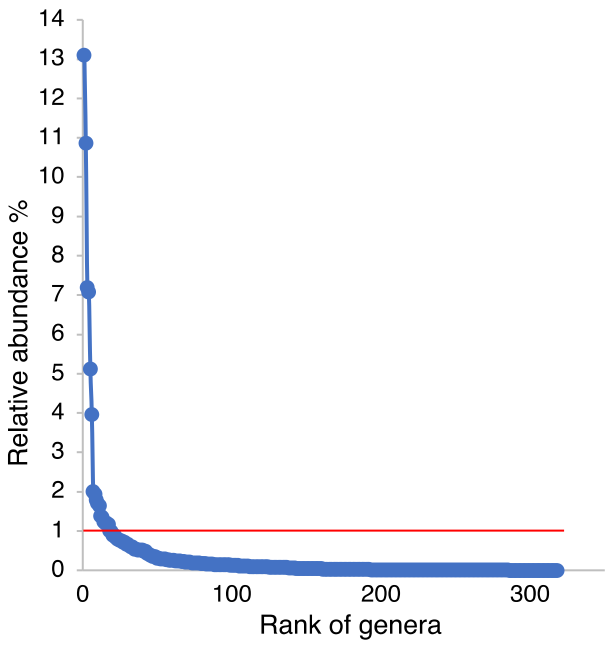

Figure S1 Rank-abundance curve of bacterial genera to determine rare genera cut-off. Red line shows the curve’s approximate inflection at 1 % relative abundance.

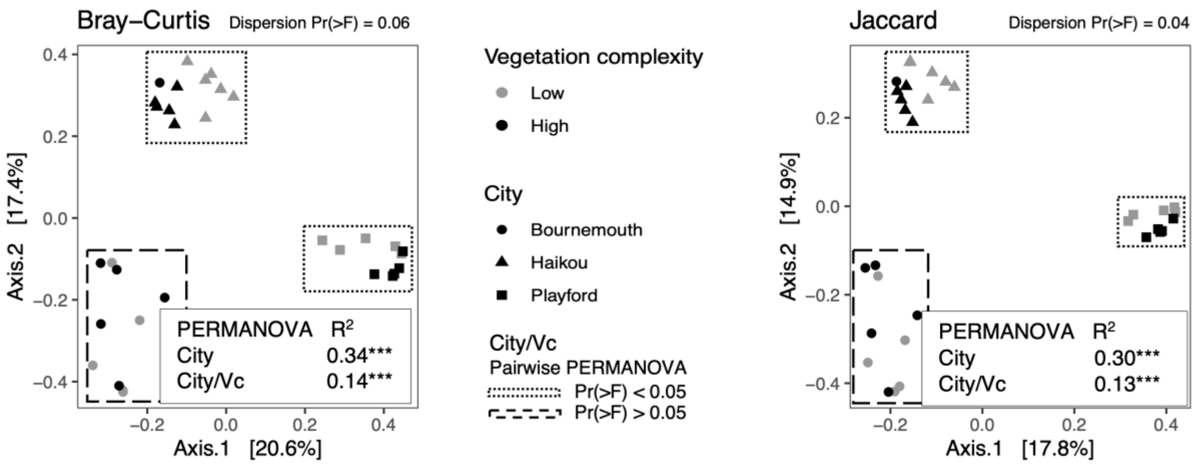

Figure S2 ASV-level ordinations. Jaccard is over-dispersed and Bray-Curtis is very close to it.

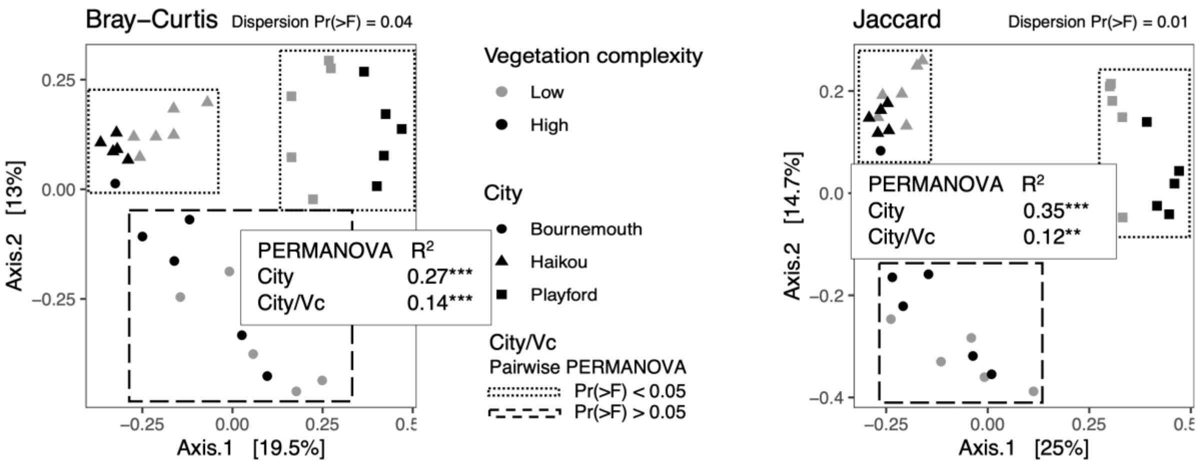

Figure S3 Ordinations of rare genera only (those < 1 % relative abundance). Both datasets are over-dispersed.
